# Improvements in body composition, functional capacity, and cardiovascular fitness: results of a workplace wellness program in the United Arab Emirates

**DOI:** 10.3389/fspor.2026.1726817

**Published:** 2026-03-23

**Authors:** Emad Masuadi, Alshafi Mohammad, Salma Al Hosani, Erik Koornneef, Javaid Nauman

**Affiliations:** 1 Institute of Public Health, College of Medicine and Health Sciences, United Arab Emirates University, Al-Ain, United Arab Emirates; 2 Clinical Trials Unit, Sheikh Shakhbout Medical City, Abu Dhabi, United Arab Emirates; 3 Research and Education, The Medical Office (a Pure Health Company), Dubai, United Arab Emirates; 4 Health Operations Management, Ambulatory Health Services, SEHA, Abu Dhabi, United Arab Emirates; 5 Department of Circulation and Medical Imaging, Faculty of Medicine and Health Sciences, Norwegian University of Science and Technology (NTNU), Trondheim, Norway; 6 Healthy Living for Pandemic Event Protection (HL-PIVOT) Network, Chicago, IL, United States

**Keywords:** body mass index, cardiorespiratory fitness, exercise, prevention, waist circumference, wellness programs, workplace

## Abstract

**Introduction:**

Chronic diseases are the largest contributor to overall morbidity and mortality. The workplace lifestyle interventions have shown improvements in anthropometric, and cardiometabolic parameters. However, data related to workplace wellness programs in the United Arab Emirates (UAE) are scarce. The aim of this study was to evaluate the effectiveness of a workplace wellness intervention on anthropometric measures, functional capacity and cardiovascular fitness.

**Methods:**

We conducted a 12-week workplace wellness intervention among employees of a leading UAE healthcare organization. The program included physical activity challenges targeting step count and calorie expenditure, with assessments conducted at baseline, day 45, and day 90. We used adjusted linear mixed-effects models to analyse the data.

**Results:**

A total of 116 participants were included in the analysis [mean age 39.2 (SD 8.4) years, female sex 49.1%]. Compared with baseline values, we observed a significant improvement in weight, body mass index, and waist circumference both at day 45 [−0.52 (95% CI, −0.96 to −0.08) kg, −0.18 (−0.32 to −0.03) kg/m^2^, −2.53 (−3.93 to −1.14) cm, respectively], and at day 90 [−1.16 (−1.81 to −0.51) kg, −0.40 (−0.62 to −0.18) kg/m^2^, −3.73 (−5.84 to −1.59) cm, respectively]. The total functional movement score increased by 2.76 (2.23–3.30) and 4.99 (4.50–5.49) at day 45 and day 90, respectively. The systolic blood pressure was decreased by −2.58 (−5.09 to −0.07) mmHg and −4.76 (−7.09 to −2.43) mmHg, and Rockport 1-mile walking time was decreased by −2.06 (−2.57 to −1.55) minutes and −2.46 (−3.11 to −1.82) minutes at day 45 and day 90, respectively, compared with the baseline values. The predicted cardiorespiratory fitness (VO_2_) increased by 9.00 (7.28–10.73) mL·kg^−1^·min^−1^ at day 45, and by 7.24 (5.34–9.13) mL·kg^−1^·min^−1^ at day 90. Compared with week-1 levels, the activity wearable parameters of steps and calories per day showed reductions mid-intervention [−1,392 steps/day, (−2,233 to −550); −248 calories/day, (−404 to −92)], and by day 90 [−1,008 steps/day (−2,164 to 148); −157 calories/day (−310 to −5). The wearable-derived resting heart rate showed a modest decline with mean reductions of −1.41 bpm (−2.52 to −0.30) at mid-intervention, and −0.57 bpm (−1.79 to 0.66) by day 90.

**Discussion:**

Our findings show significant improvements in anthropometry, functional movement scores, and selected parameters of cardiovascular fitness associated with workplace wellness program. These results contribute important preliminary data for the UAE healthcare workforce, and suggest that incorporating workplace interventions into organizational health policies could play a crucial role in improving employee health.

## Introduction

Chronic diseases, namely diabetes, cardiovascular disease, cancer, and chronic respiratory diseases are leading contributors to morbidity and premature mortality worldwide, accounting for over 70% of all deaths (1). On the other hand, modifiable risk factors encompassing behavioural, environmental, occupational, and metabolic risk factors are powerful tools for prevention of diseases and injuries. In 2021, 41% of global disability adjusted life years (DALYs) were attributed to these risk factors (2). Importantly, the metabolic risk factors, in particular elevated systolic blood pressure, increased body mass index (BMI), and high fasting plasma glucose, significantly contribute to the attributable disease burden, and if left untreated, these factors are anticipated to increase the mortality rates in the future (2, 3).

The United Arab Emirates (UAE) has experienced a notable increase in demand for healthcare services due to rapid population growth, a rise in non-communicable diseases, and a progressively ageing population (4). In recent years, the prevalence of chronic diseases associated with unhealthy lifestyle has increased in the UAE, and the data in the adult population from 2017 to 2018 suggest that 68% are overweight, 28% are obese, 29% have high blood pressure, 44% have high cholesterol, and 12% have diabetes (5). The high disease burden translates into increased healthcare expenditures, and chronic diseases cost the UAE economy an approximate US$ 10.9 billion annually (6).

The Sustainable Development Goals (SDGs) include a target to reduce premature mortality from chronic diseases by one-third by 2030 (7). To achieve this target, purposefully designed cost-effective workplace-based (non-clinical settings) interventions which specifically target the modifiable risk factors with a focus on enhancing overall population health and reducing the risk of chronic diseases are recommended (8, 9). In this regard, workplace-or-community-based lifestyle interventions have shown improvements in the body weight, dietary measures, cardiometabolic health, and health related quality of life (10–13). For example, the results of a systematic review and meta-analysis of workplace wellness programs showed improvements in body mass index (−0.22 kg/m^2^), waist circumference (−1.47 cm), body weight (−0.92 kg), and blood pressure parameters (10). Consistent with these findings, physical activity and wellness programs in public sector and healthcare employees in diverse settings have reported higher physical activity levels, improved cardiorespiratory fitness, and favourable changes in cardiometabolic risk and work-related outcomes (11, 13–15).

In the Gulf Cooperation Council (GCC) region, including the UAE, workplace wellness initiatives are gaining momentum, and recent evidence demonstrates that workplace physical activity interventions can achieve meaningful increases in daily step counts, and moderate-to-vigorous physical activity (16, 17). The World Health Organization and United Nations Interagency Task Force on NCDs in collaboration with UAE Ministry of Health, advocates for strategic investments in clinical and population-based interventions aimed at the prevention and control of chronic diseases in the UAE (6). Despite this, limited research has specifically examined workplace interventions targeting body composition, functional capacity, and cardiovascular fitness in the UAE context. Functional capacity, defined as an individual's ability to perform physical activities and job-related tasks safely and efficiently, represents an important health outcome that bridges physical fitness with occupational performance. This parameter includes strength, endurance, flexibility, and coordination which are attributes essential for both workplace productivity and daily living activities (18). Lower functional capacity and musculoskeletal pain have been linked to higher rates of work disability, sickness absence, and productivity across a range of occupations, including healthcare and office-based workers (19–21). On the other hand, workplace exercise interventions that improve functional capacity can reduce musculoskeletal pain and may enhance workers' health, efficiency, and productivity (19). Therefore, improving functional capacity through workplace wellness programs may provide benefits for employee health and organizational occupational health and safety outcomes.

The aim of this paper is to present the outcomes of a 90-day workplace wellness program on body composition parameters, functional capacity measures, and cardiovascular fitness indicators among 116 adult employees in the UAE. We hypothesized that a 90-day workplace wellness program would significantly improve body composition, functional capacity, and cardiovascular fitness among UAE healthcare employees. Our study addresses a critical gap in the regional literature by providing empirical evidence for workplace wellness intervention effectiveness in the UAE context, contributing valuable insights for public health policy and occupational health practice in the Gulf region.

## Methods

### Study settings and participants

The study was single-arm workplace intervention designed as an exploratory pilot study among the employees of one of the largest health providing companies in the UAE. A detailed account of study including participants recruitment, eligibility, and data collection has been described elsewhere (22). In brief, employees were eligible to participate in the study If they were 18 years and older, available during the study duration, willing to commit to all the procedures and activities relevant to the study during its three months period, and confirm participation through the informed consent form. The exclusion criteria included: unsure or unable to fully commit to all the procedures and activities relevant to the study; advised not to exercise by a licensed healthcare practitioner; pregnant or breastfeeding; severe illnesses; severe injury in the joints or the back; planning major surgical procedures or other major treatment during the study period; or participating in any other health promotion program.

Participants were recruited through an online invitation which was sent to 1,859 employees, of which 15% (*n* = 279) volunteered to participate. From these volunteers, 116 participants were selected to ensure a representative distribution of sex and ethnicity in alignment with the national demographics. The final sample included individuals from a diverse range of professional roles, such as medical staff, allied health professionals, administrative and technical personnel, as well as those in human resources, finance and accounting, marketing and public relations, legal and compliance, management, and executive positions (22). The analytical sample size is consistent with recommendations for pilot and feasibility studies designed to inform the planning of subsequent trials (23, 24).

#### Data collection

Trained personnel including registered nurses, licensed nutritionist, certified fitness instructors collected the relevant data using the standardized procedures. Detailed accounts are described elsewhere (22). Below is a brief summary of data collection process.

### Body composition

Body weight and height were measured in kilograms and centimetres using a weighing scale and stadiometer, and body mass index (BMI) was calculated by dividing weight in kilograms by the square of height in meters (kg/m^2^). A non-elastic measuring tape was used to measure the waist circumference in centimetres, and body fat percentage was measured using a handheld body fat analyser.

### Functional capacity

Functional capacity was assessed using a Functional Movement Screen (FMS) test kit, which employs a simple grading system to evaluate movement quality. The test included deep squats, hurdle steps, in-line lunges, shoulder mobility, active straight leg raises, trunk stability push-ups, and rotary stability. Scores ranged from 0 to 3: 0 indicated pain during movement; 1, inability to perform or achieve correct positioning; 2, completion with compensation; and 3, optimal execution without compensation. Total scores ranged from 0 to 21, with scores ≤14 indicating a 1.5–2.0 times higher injury risk (25). Additional dichotomous variables, such as the shoulder extension, and flexion clearing tests, were scored as positive or negative. The ankle clearing test was categorized as beyond, within, or behind, while areas for improvement were classified as either mobility or stability.

### Cardiovascular fitness

Cardiovascular fitness was evaluated through a combination of numerical and categorical variables. The numerical variables included systolic and diastolic blood pressure, resting and exercise heart rates, 1-mile Rockport walking test results, predicted peak oxygen consumption (VO_2_), and fitness percentile. Blood pressure was measured with a mobile monitor, categorizing results as normal (<120/80 mm Hg), pre-hypertension (120–139/80–89 mm Hg), or hypertension (≥140/90 mmHg). Resting and exercise heart rates (measured at the start and end of the 1-mile Rockport test, respectively) were recorded in beats per minute (bpm) using a heart rate monitor. The 1-mile Rockport test was conducted on a commercial treadmill, with completion time recorded in minutes and seconds using a stopwatch after a 5–10-minute warm-up. Predicted VO_2_ (mL·kg^−1^ min^−1^) was calculated to estimate aerobic capacity based on age, sex, body weight, walking completion time, and exercise heart rate, using the following formula (26):

$$\begin{array}{rl} {\mathrm{VO}}_{2},\;\mathrm{mL}\cdot {\mathrm{kg}}^{-1}\cdot \min^{-1} & =132.853\;-(0.0769\times \mathrm{weight}\_\mathrm{lb})\\ & \;-(0.3877\times \mathrm{age}\_\mathrm{years})+(6.315\times \mathrm{sex})\\ & \;-(3.2649\times \mathrm{time}\_\mathrm{minutes})\;,\\ & \;-(0.1565\times \mathrm{heartrate}\_\mathrm{bpm}), \end{array}$$

where male = 1 and female = 0. Body weight was converted from kilograms to pounds (lb).

#### Workplace wellness program

After the baseline assessment, participants were asked to download and sign in to the mobile application, which was the primary tool of intervention and data collection. The app was integrated with the Fitbit fitness trackers of participants in order to track physical activity and monitor program adherence. During the intervention period, participants received regular notifications, details about the program, and a reminder of upcoming challenges through the mobile app.

The intervention was designed as a 12-week workplace wellness program (December 2022—March 2023) involving progressive physical activity and health behaviour challenges. Participants were invited to participate in both individual and corporate challenges to increase daily step counts, caloric expenditure, and sleep duration. Challenges included the following:

Weeks 1–2: Step count challenges, with a minimum of 8,000 daily steps in week 1 and a build-up to 9,000 daily steps in week 2.

Weeks 3–4: Caloric burn targets, set at ≥2,000 kcal/day for women and ≥3,000 kcal/day for men with daily incremental increases.

Weeks 5–6: Weekly step count targets starting with a minimum of 8,000 steps in week 5 and increasing to 8,500 steps in week 6.

Weeks 7–8: Re-introduce caloric burn goals with week 8 having a secondary daily step goal of 10,000 steps.

Weeks 9–11 (Multi-tier challenges): Step and caloric burn challenges with tiered options to allow for individual differences in fitness levels. For example, participants could choose between step targets of 6,000 or 10,000 steps/day and caloric burn goals ranging from ≥1,500 to ≥3,000 kcal/day.

Week 12: Final caloric burn challenge, set at ≥2,000 kcal/day for females and ≥3,000 kcal/day for males.

Participants were encouraged to achieve the weekly step and caloric targets primarily through activities of at least moderate intensity (e.g., brisk walking, stair climbing, cycling, circuit-type resistance exercise) in line with adult physical activity recommendations. Adherence was supported through app-based notifications and reminders, visible progress dashboards, and corporate challenges, including leader boards. Brief educational messages on physical activity and recovery were also delivered through the app during the 12-week program.

#### Ethics approval and consent to participate

This study was approved by the Institutional Review Board (IRB) of Abu Dhabi Health Services Company (SEHA) under the application number SEHA-IRB-45, and performed in accordance with relevant guidelines and regulations including the Declaration of Helsinki. Informed consent was obtained from all the participants.

#### Statistical analyses

The data were analysed using the statistical software IBM SPSS Statistics for Windows, Version 29, Armonk, NY: IBM Corp. The baseline characteristics of the participants were presented as frequencies and percentages for categorical variables, as well as means (SD) for continuous variables. For anthropometric, FMS, cardiovascular related outcomes, changes over time at baseline, 45 days, and 90 days were analysed using linear mixed-effects models adjusted for age, sex, smoking status, physical activity, and included as fixed effect factors. Model assumptions were verified, including normality of residuals which were confirmed graphically using Q-Q plots. The random intercepts for participants were used to account for heterogeneity, and an unstructured covariance matrix was applied to accommodate the correlation between repeated time measurements. For Fitbit outcomes of daily values of steps, calories, sleep hours, and resting heart rate, we defined three 7-day windows: week 1 (days 1–7; used as reference), middle (days 42–48), and pre-day-90 (days 84–90), to align with clinical assessments. The daily records were filtered by wear-time proxy (steps≥100), and window means required ≥5 valid days. The linear mixed-effects models for Fitbit data additionally included fixed effect of window+age, sex, smoking status, and physical activity. All tests were performed at a 5% significance level.

## Results

The mean age of the participants at baseline was 39.2 (SD 8.4) years, and almost half of them were women (49.1%). Around two-thirds of the participants were non-smokers, and more than 90% had a bachelor degree or higher education. The majority of participants (88.4%) reported no chronic disease (Table 1). Among 116 participants with completed baseline data assessments, nine (7.8%) were lost to follow-up at day 90.

**Table 1:** Baseline characteristics of the participants.

Variables		N	%
Age (years)	<30	17	14.7
	30–39.9	50	43.1
	40–49.9	35	30.2
	50+	14	12.1
Mean Age (SD), years	39.2 (8.4)	116	100
Sex	Female	57	49.1
Nationality	Emirati	23	19.8
	Non-Emirati	93	80.2
Ethnicity	Middle-Eastern-Arab	34	29.3
	Pakistani-Indian	33	28.4
	Asian	25	21.6
	White-Caucasian	17	14.7
	Black/African/Caribbean	6	5.2
	Latin-American-Hispanic	1	0.9
Marital Status	Single	34	29.3
	Married	73	62.9
	Divorced	8	6.9
	Widowed	1	0.9
Children	0	38	33.9
	1	22	19.6
	2	31	27.7
	3	13	11.6
	More than 3	8	7.1
Education Level	High school certificate	2	1.8
	Bachelor's degree	51	45.5
	Master's-degree	48	42.9
	Ph.D.	5	4.5
	Other	6	5.4
Do you smoke?	No	77	68.8
	Occasionally	19	17.0
	Yes	16	14.3
How often do you exercise?	Never	9	8.1
	Less-often	16	14.4
	Once-per-week	18	16.2
	Several-times-per-month	14	12.6
	Every other day	41	36.9
	Everyday	13	11.7
Do you currently live with any chronic diseases?	No	99	88.4
	Yes	13	11.6
On a scale from 1 to 10, how important do you think this initiative is? (10 being extremely important)	5	3	2.7
	6	1	0.9
	7	3	2.7
	8	12	10.7
	9	15	13.4
	10	78	69.6
On a scale from 1 to 10, how likely are you to commit to and complete the 3-month Longevity trial? (10 being extremely likely)	3	1	0.9
	5	1	0.9
	6	4	3.6
	7	4	3.6
	8	15	13.4
	9	19	17.0
	10	68	60.7

For body composition outcomes such as weight, BMI, waist circumference, there were significant improvements at both day 45 and day 90 (Table 2). The waist circumference decreased by an average of 2.53 cm (95% CI, 1.14–3.93), and by 3.73 cm (95% CI, 1.59–5.84) at days 45 and 90, respectively, compared with the baseline values. Similar trends were observed for body weight and BMI, however, no significant differences were found for body fat percentage, although, there was a decreasing trend both at day 45 and day 90 (Table 2).

**Table 2:** Change in the body composition parameters.

Measurement		Mean	95% CI
Weight (kg)	Baseline value	81.42	(76.93–85.90)
	Difference at 45 days	−0.52*	(−0.96 to −0.08)
	Difference at 90 days	−1.16*	(−1.81 to −0.51)
	Difference 45 vs. 90 days	−0.64*	(−1.03 to −0.26)
Body Mass Index (kg/m^2^)	Baseline value	27.94	(26.42–29.45)
	Difference at 45 days	−0.18*	(−0.32 to −0.03)
	Difference at 90 days	−0.40*	(−0.62 to −0.18)
	Difference 45 vs. 90 days	−0.23*	(−0.36 to −0.10)
Waist Circumference (cm)	Baseline value	94.34	(90.75–97.93)
	Difference at 45 days	−2.53*	(−3.93 to −1.14)
	Difference at 90 days	−3.73*	(−5.84 to −1.59)
	Difference 45 vs. 90 days	−1.18	(−3.07 to 0.70)
Body Fat Percentage (%)	Baseline value	29.05	(27.05–31.05)
	Difference at 45 days	−0.42	(−1.10 to 0.26)
	Difference at 90 days	−0.55	(−1.29 to 0.18)
	Difference 45 vs. 90 days	−0.13	(−0.84 to 0.58)

Data are estimated mean and 95% confidence intervals (CI).

* *P* < 0.05.

The individual components of FMS test showed improvements both at day 45 and day 90 measurements with higher scores compared with the baseline. Accordingly, the total FMS score increased by 2.76 (95% CI, 2.23–3.30) and 4.99 (95% CI, 4.50–5.49) at day 45 and day 90, respectively, compared to baseline values (Table 3).

**Table 3:** Change in the functional fitness parameters.

Measurement		Mean	95% CI
Deep Squat Final Score	Baseline value	1.87	(1.70–2.04)
	Difference at 45 days	0.53*	(0.38–0.68)
	Difference at 90 days	0.85*	(0.72–0.99)
	Difference 45 vs. 90 days	0.32*	(0.21–0.44)
Hurdle Step Final Score	Baseline value	1.54	(1.40–1.68)
	Difference at 45 days	0.35*	(0.21–0.49)
	Difference at 90 days	0.94*	(0.80–1.07)
	Difference 45 vs. 90 days	0.59*	(0.46–0.72)
In Line Lunge Final Score	Baseline value	1.56	(1.41–1.72)
	Difference at 45 days	0.53*	(0.34–0.71)
	Difference at 90 days	0.92*	(0.78–1.05)
	Difference 45 vs. 90 days	0.39*	(0.22–0.56)
Shoulder Mobility Final Score	Baseline value	1.93	(1.72–2.14)
	Difference at 45 days	0.38*	(0.17–0.58)
	Difference at 90 days	0.53*	(0.36–0.70)
	Difference 45 vs. 90 days	0.15*	(0.01–0.30)
Trunk Stability Push-Up Final Score	Baseline value	1.64	(1.48–1.79)
	Difference at 45 days	0.80*	(0.63–0.96)
	Difference at 90 days	0.91*	(0.75–1.07)
	Difference 45 vs. 90 days	0.11*	(0.00–0.23)
Rotatory Stability Final Score	Baseline value	1.22	(1.08–1.37)
	Difference at 45 days	0.65*	(0.50–0.81)
	Difference at 90 days	0.85*	(0.69–1.00)
	Difference 45 vs, 90 days	0.19*	(0.07–0.31)
FMS Total Score (0**–**21)	Baseline value	11.69	(11.01–12.37)
	Difference at 45 days	2.76*	(2.23–3.30)
	Difference at 90 days	4.995*	(4.50–5.49)
	Difference 45 vs, 90 days	2.232*	(1.75–2.71)

Data are estimated mean and 95% confidence intervals (CI).

* *P* < 0.05.

For selected parameters of cardiovascular fitness, systolic blood pressure decreased by 2.58 mmHg and 4.76 mmHg at day 45 and day 90, respectively, compared with the baseline values. Similar improvements were observed in diastolic blood pressure (−0.93 mmHg at day 45, −2.07 mmHg at day 90), and Rockport walk completion time (−2.06 min at day 45, −2.46 min at day 90). At day 45, predicted VO_2_ increased by 9.00 mL.kg^−1^.min^−1^, compared with the baseline value, and at day 90, an improvement of 7.24 mL.kg^−1^.min^−1^ was observed (Table 4).

**Table 4:** Change in the cardiovascular fitness parameters.

Measurement		Mean	95% CI
Systolic Blood Pressure (mmHg)	Baseline value	123.94	(120.30–127.59)
	Difference at 45 days	−2.58*	(−5.09 to −0.07)
	Difference at 90 days	−4.76*	(−7.09 to −2.43)
	Difference 45 vs. 90 days	−2.18	(−4.62 to 0.25)
Diastolic Blood Pressure (mmHg)	Baseline value	76.58	(74.09–79.07)
	Difference at 45 days	−0.93	(−2.58 to 0.72)
	Difference at 90 days	−2.07*	(−3.86 to −0.27)
	Difference 45 vs. 90 days	−1.14	(−2.87 to 0.60)
Resting Heart Rate (bpm)	Baseline value	86.66	(83.06–90.25)
	Difference at 45 days	−13.12*	(−16.87 to −9.37)
	Difference at 90 days	−14.84*	(−17.81 to −11.86)
	Difference 45 vs. 90 days	−1.71	(−4.69 to 1.26)
Walk Completion Heart Rate (mmHg)	Baseline value	131.95	(127.00–136.91)
	Difference at 45 days	2.62	(−2.18 to 7.41)
	Difference at 90 days	6.97*	(2.37–11.58)
	Difference 45 vs. 90 days	4.36*	(0.27–8.45)
Walk Completion Time in Minutes	Baseline value	18.57	(17.63–19.51)
	Difference at 45 days	−2.06*	(−2.57 to −1.55)
	Difference at 90 days	−2.46*	(−3.11 to −1.82)
	Difference 45 vs. 90 days	−0.41	(−0.88 to 0.07)
Predicted VO_2peak_ (mL·kg^−1^·min^−1^)	Baseline value	23.85	(20.54–27.17)
	Difference at 45 days	9.00*	(7.28–10.73)
	Difference at 90 days	7.24*	(5.34–9.13)
	Difference 45 vs. 90 days	−1.77*	(−3.44 to −0.10)

Data are estimated mean and 95% confidence intervals (CI).

* *P* < 0.05.

**Figure 1:**
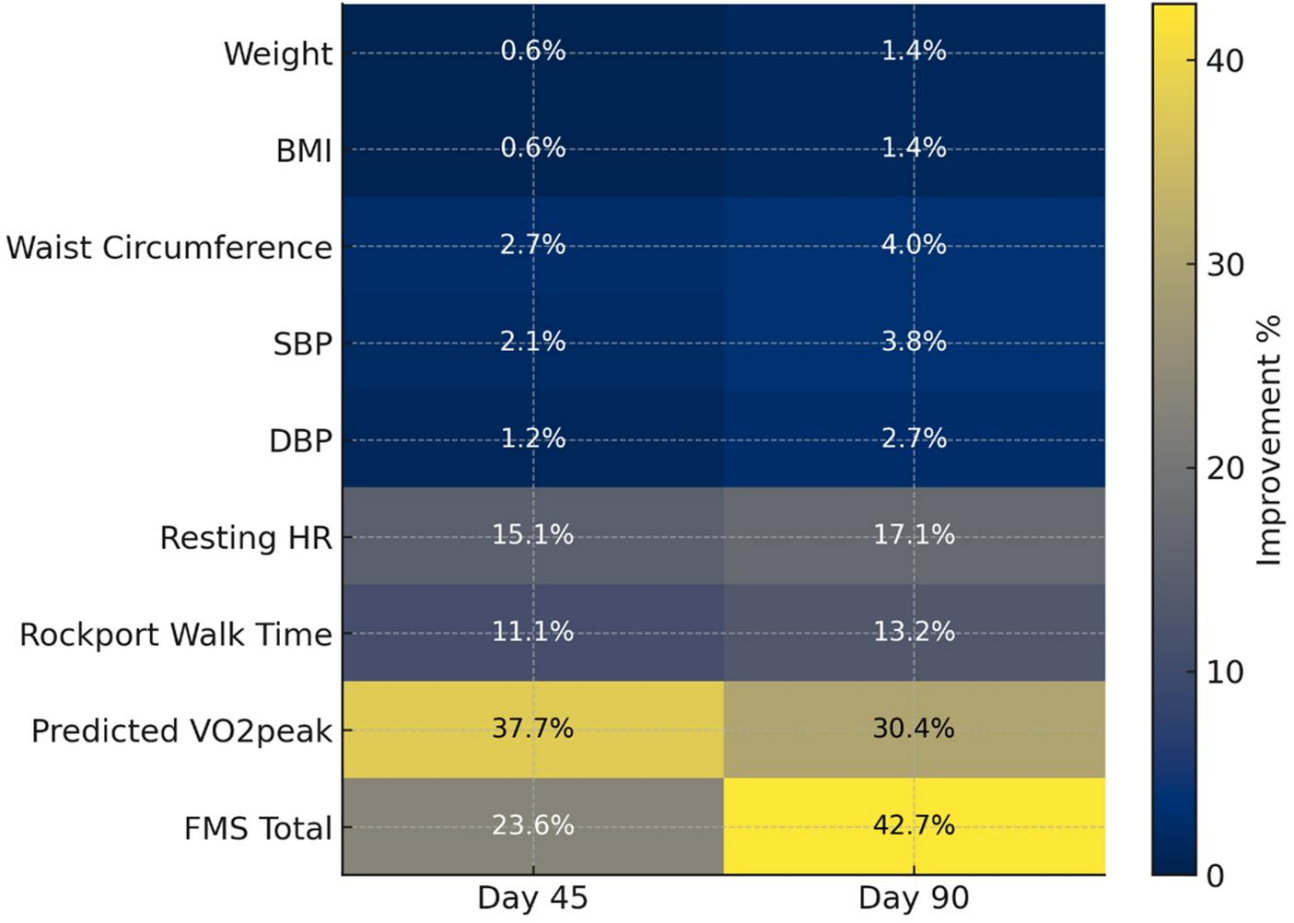
Improvements in selected parameters as percent change from baseline. Estimates derived from model-based mean differences (Tables 2–4). Positive values indicate improvement. For outcomes where lower is better [weight, body mass index—BMI, waist, systolic blood pressure—SBP, diastolic blood pressure—DBP, resting heart rate (HR), Rockport walk time]: improvement % = −Δ/baseline  × 100%. For outcomes where higher is better (functional movement score—FMS total, predicted VO₂), improvement % = Δ/baseline  × 100%.

The improvements in the clinical assessments are also shown in Figure 1 as the % change from baseline to day 45, and day 90. The positive values indicate improvements, and % change estimates were derived from model -based mean differences presented in Tables 2–4.

For Fitbit outcomes, daily mean steps count showed a dip during week 3 and week 8, and returning to or surpassing week-1 levels by day 90. The resting heart rate showed a consistent decline over the 90-day period; however, no changes were observed for sleep duration (Supplementary Figure 1 in supplementary materials). The adjusted results of linear mixed models were aligned with the descriptive analysis showing reductions in daily steps and calories; whereas resting heart rate showed improvement, which is consistent with the observed clinic-based improvements in Rockport performance, and predicted VO_2_. For example, steps/day showed reductions at middle vs. week 1 (−1,392, 95% CI, −2,233 to −550), and pre-day-90 vs. week 1 (−1,008, 95% CI, −2,164 to 148). The corresponding reductions in calories/day were −248 (95% CI, −404 to −92) at middle, and −157 (95% CI, −310 to −5) at pre-day-90. Sleep did not change materially (middle: −0.002 hours, 95% CI, −0.542 to 0.538; pre-day-90: 0.063 hours, 95% CI, −0.396 to 0.522), and resting heart rate (beats/min) showed reductions at middle (−1.41, 95% CI, −2.52 to −0.30) and at pre-day-90 (−0.57, 95% CI, −1.79 to 0.66) (Table 5).

**Table 5:** Change in the fitbit parameters (daily average per week).

Measurement		Mean	95% CI
Steps count	Week 1	10,679.1	(9,404.6–11,953.5)
	Difference at middle	−1,391.9*	(−2,233.4 to −550.3)
	Difference at final week	−1,008.3	(−2,164.3 to 147.6)
	Difference middle vs final week	383.5	(−1,502.0 to 735.0)
Sleeping hours	Week 1	6.59	(6.11–7.08)
	Difference at middle	−0.002	(−0.542 to 0.538)
	Difference at final week	0.063	(−0.396 to 0.522)
	Difference middle vs final week	0.065	(−0.219 to 0.348)
Calories burned	Week 1	2,691.9	(2,476.4–2,907.4)
	Difference at middle	−248.2*	(−404.4 to −91.9)
	Difference at final week	−157.3*	(−309.6 to −5.1)
	Difference middle vs final week	90.8	(−28.0 to 209.7)
Resting Heart Rate	Week 1	69.8	(67.3–72.4)
	Difference at middle	−1.41*	(−2.52 to −0.30)
	Difference at final week	−0.57	(−1.79 to 0.66)
	Difference middle vs final week	0.85	(−0.22 to 1.92)

Data are estimated means and 95% confidence intervals (CI).

We defined three 7-day windows: week 1 (days 1–7; used as reference), middle (days 42–48), and final week—pre-day-90 (days 84–90), to align with clinical assessments.

* *P* < 0.05.

## Discussion

The results of this study showed that a 90-day workplace wellness program resulted in significant improvements in body weight, waist circumference, functional capacity, and cardiovascular fitness including decrease in blood pressure, increases in exercise heart rate and cardiorespiratory fitness.

The results of our study are consistent with the findings of a recent meta-analysis (10), and earlier studies (27–34) showing that workplace wellness programs are associated with improvements in anthropometric and cardiometabolic factors. The results of meta-analysis showed significant decreases in BMI (−0.22 kg/m^2^), body weight (−0.92 kg), waist circumference (−1.47 cm), and systolic blood pressure (−2.03 mmHg) associated with multi-component workplace wellness programs (10). Previous studies of workplace wellness programs have reported effect sizes ranging between −5.90 and 0.10 cm for waist circumference (10, 27, 31), −8.90 and 0.00 kg for body weight (33, 35, 36), −3.10 and 0.00 kg/m^2^ for BMI (27, 35, 36), and −15.00 and 0.20 mmHg for systolic blood pressure (30, 34, 35, 37). We observed a 4.0% decrease (−3.73 cm) in waist circumference, 1.4% decrease in both body weight (−1.16 kg) and BMI (−0.40 kg/m^2^), 3.8% decrease (−4.76 mm HG) in systolic blood pressure, and 2.7% decrease (−2.07 mmHg) in diastolic blood pressure at 90 days. The differences in results across studies may be due to different study designs (RCTs, quasi-experimental studies, experimental studies without comparison groups or observational), baseline populations and settings, and different components of the workplace wellness programs. In clinical context, a 5% reduction in body weight (38) is considered meaningful, and a 5 mmHG reduction in systolic blood pressure (39) is associated with lower incidence of cardiovascular events. However, the probability of 5% or greater weight loss among overweight and obese population is about 10% (40), and interventions or wellness programs at workplaces can be used as key strategies for improving population health (8, 10).

In contrast to the significant reductions in body weight, BMI and waist circumference, body fat percentage showed only a non-significant downward trend at 45 and 90 days. These results may reflect limited sensitivity and between-day variability of the handheld body fat analyser, the modest absolute weight loss, and the relatively short intervention duration (41, 42).

Our results showed an improvement in the individual components FMS scores, and the total FMS score increased by 42.7% at the 90-day measurements. Earlier studies have shown that workplace interventions are associated with reduction of musculoskeletal discomfort (43), improvements in low back pain (44), a beneficial effect in reducing shoulder pain intensity (45), and overall improvements in muscular endurance and muscle power (46, 47).

For the selected parameters of cardiovascular fitness, we observed improvements in heart rates at rest and during exercise, and predicted values of VO_2_ (46, 48, 49). Previous studies of workplace exercise RCTs have shown beneficial improvements in the VO_2_. In a meta-analysis of 12 studies (48), workplace physical activity interventions were associated with 2.7 mL·kg^−1^·min^−1^ higher VO_2_, and the effect estimates ranged from −2.0 to 8.0 mL·kg^−1^·min^−1^ (50–52). Our estimates of VO_2_ improvement at 90 days (7.24 mL·kg^−1^·min^−1^) are within the range of what is reported in earlier studies, and emphasize that workplace wellness programs with physical activity component can be used as an effective strategy to improve cardiorespiratory fitness which maybe the single best predictor for mortality and survival (53). The observed reductions in resting heart rate during clinical assessments are consistent with the improvements in Rockport performance and predicted VO_2_, and with evidence that aerobic exercise training lowers resting heart rate, and that resting heart rate is a population-level biomarker of cardiorespiratory fitness (54, 55). However, the magnitude of change in resting heart rate should be interpreted with caution, as it may partly reflect measurement context, regression to the mean, and individual variability in autonomic adaptation.

We observed a decrease in daily step count and calorie expenditure, while resting heart rate recorded via wearable devices decreased compared with the week 1 measurements. The baseline (day 0 or prior) measurements of the wearable data were not available, and week 1 recordings which is also the first week of the active intervention were used as the reference group for comparison at middle (days 42–48), and pre-day-90 (days 84–90), to align with clinical assessments. The observed decline in step count and calorie expenditure in our study from week 1 to later timepoints is in line with well-documented patterns in exercise intervention research. The findings of previous studies show that intervention adherence decreases over time across diverse populations and intervention types (56–58). Data also show that motivation to exercise naturally decreases over time, with dropout rates ranging from 7% to 58%, and significant declines in exercise participation as intervention duration increases (59–61). Nevertheless, a significant lower dose of daily steps has been reported to be associated with clinically meaningful improvements in health outcomes: 7,000 steps per day (62); with optimal doses (maximal risk reductions) of 8,763 steps/day for all-cause, and 7,126 steps/day for cardiovascular outcomes (63). Interestingly, the steps/day above the optimal dose did not show any additional reductions in mortality and cardiovascular risk (63). Another likely explanation for the reduced step volume despite clinical improvements could be intervention's weekly challenge structure, which alternated between step-focused and calorie-focused goals. For example, when weekly challenge was calorie goal, participants could meet the targets through non-ambulatory or higher intensity activities such as cycling, resistance circuit or other structured exercises that require fewer steps. According to the 2011 Compendium of Physical Activities (64), stationary cycling at moderate-to-vigorous effort (∼6.8 METs), vigorous circuit training (∼8.0 METs), and resistance training (3.5–6.0 METs) can provide substantial energy expenditure while contributing minimal step counts compared to walking activities (4.3–5.0 METs). Moreover, consistent decline is resting heart rate throughout the intervention period aligns with the observed improvements in VO_2_, and represents a well-established physiological adaptation to exercise training (65). Resting heart rate serves as a valid population-level biomarker of cardiorespiratory fitness, and exercise training reduces resting heart rate across diverse populations, with the magnitude of reduction correlating with improvements in the fitness (54, 55).

Collectively, our results demonstrate physiological and performance adaptations that occurred independently of sustained increases in step volume, reflecting a phenomenon where individuals often reduce incidental physical activity in favour of structured behavioural substitutions during intervention periods. This observation is consistent with the Constrained Total Energy Expenditure model (66, 67), which states that total daily energy plateaus as activity rises, with trade-offs across domains rather than linear add-ups. Earlier studies have also documented that increases in structured exercise can result in compensatory decreases in non-exercise activity thermogenesis (NEAT), including reduced spontaneous movement and incidental activities throughout the day (68–70). In our study, this framework provides a plausible explanation for why wearable-derived step counts and calories decreased from week 1 while clinic-based markers of fitness and cardiometabolic health improved, suggesting that participants may have shifted towards more structured, higher-intensity activity patterns that are not fully captured by step volume alone. Similar dissociations between device-measured steps and physiological adaptations have been described in workplace exercise trials, where supervised or structured sessions improved VO₂ and musculoskeletal outcomes without necessarily producing sustained increases in daily step counts (48, 71).

Our findings are broadly consistent with workplace exercise trials among healthcare and other occupational groups, where supervised or structured exercise at work has prevented deterioration in work ability, improved musculoskeletal outcomes, and increased cardiorespiratory fitness (48, 71). These observations reinforce the potential for pragmatic, workplace-embedded physical activity programs to benefit relatively young, occupationally active employees, including healthcare workers.

### Strengths and limitations

The strengths of the present study include comprehensive and standardized assessments of anthropometric, functional capacity, and cardiovascular parameters. A wellness program designed for the workplace setting means the results are highly relevant to the employee health initiative and workplace wellness programs. However, several limitations must be acknowledged. Since our study was a single-arm trial without a control group, comparison between the intervention and non-intervention groups could not be considered; thus, this limits the causal inferences. We used a convenience sampling approach that may introduce selection bias, and participant's recruitment may not be fully representative of employees across the broader organization or the UAE healthcare sector. The study selection process is also prone to healthy volunteer bias, where participants who chose to join might have been more health-conscious or had a greater motivation for wellness improvement. This could lead to an overestimation of the intervention's effectiveness. We estimated the fitness based on 1-mile Rockport prediction equation rather than direct cardiopulmonary exercise testing, and the equation has not been specifically validated in UAE healthcare workers. This submaximal field test was selected for its practicality and safety in a workplace setting, and the results should be interpreted as the changes in predicted fitness rather than directly measured VO_2max_. Composite FMS scores summarize movement quality but the construct validity of FMS as a direct measure of mobility or injury risk remains debated, and scores can be influenced by factors beyond joint mobility alone (72). In addition, body fat percentage from a handheld device and free-living wearable metrics (steps, calories, resting heart rate) are subject to biological and technical variability, which may attenuate or inflate small changes over a 90-day period. Furthermore, the relatively short duration of the study might not capture long-term outcomes of the wellness program. Future studies should aim to address these limitations by incorporating randomized controlled trials, and longer follow-up periods to assess the sustainability of observed effects.

From an organisational perspective, our results support several pragmatic strategies for implementation. Employers could offer brief, protected time during shifts for structured moderate-to-vigorous activity, provide access to basic on-site or near-site exercise facilities, and deploy simple digital platforms that integrate activity tracking with progressive, gamified challenges. Embedding such initiatives within occupational health policies and leadership-endorsed wellness programs may facilitate sustained participation and help translate short-term physiological gains into longer-term reductions in modifiable cardiometabolic risk among employees (48, 71).

## Conclusion

In this single arm workplace wellness program among the UAE healthcare workforce, participants showed meaningful improvements in body weight, waist circumference, functional capacity, and cardiovascular fitness over 90 days. These preliminary data suggest that pragmatic wellness initiatives at workplaces can shift modifiable risk over a short timeframe. For practice and policy, employers and regulators could pilot and scale structured wellness policies to promote sustained benefits, e.g., protected activity time, supportive facilities, and simple objective tracking. Because this was a non-randomized, single-arm study, the findings should be confirmed in larger, randomized or stepped-wedge trials with longer follow-up to assess durability and reach across diverse roles and shifts. If replicated at scale, such programs may be a practical lever for improving workforce health in the region.

## Data Availability

The raw data supporting the conclusions of this article will be made available by the authors, without undue reservation.
