# Supplementary material for: Improvements in body composition, functional capacity, and cardiovascular fitness: results of a workplace wellness program in the United Arab Emirates
Source: Front Sports Act Living. 2026 Mar 23;8:1726817. doi: 10.3389/fspor.2026.1726817 (PMC13050850; doi:10.3389/fspor.2026.1726817)
Supplement: Supplementary file 1 [file Image1.pdf]

Supplementary Material

Figure S1. Fitbit parameter of step count, sleep hours, calories, and resting heart rate.

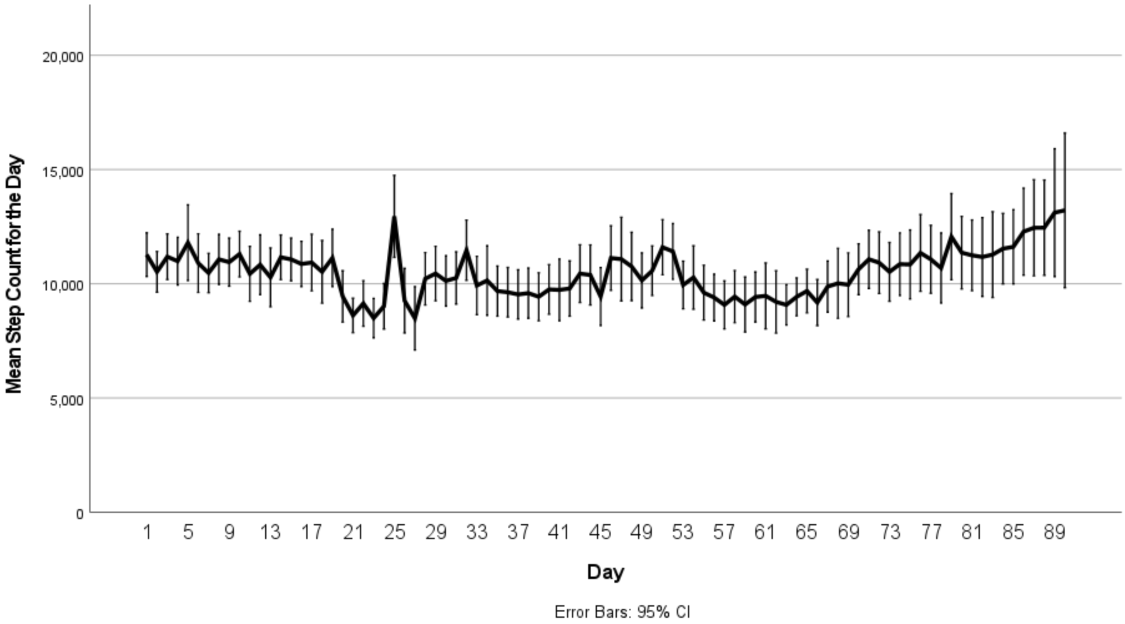

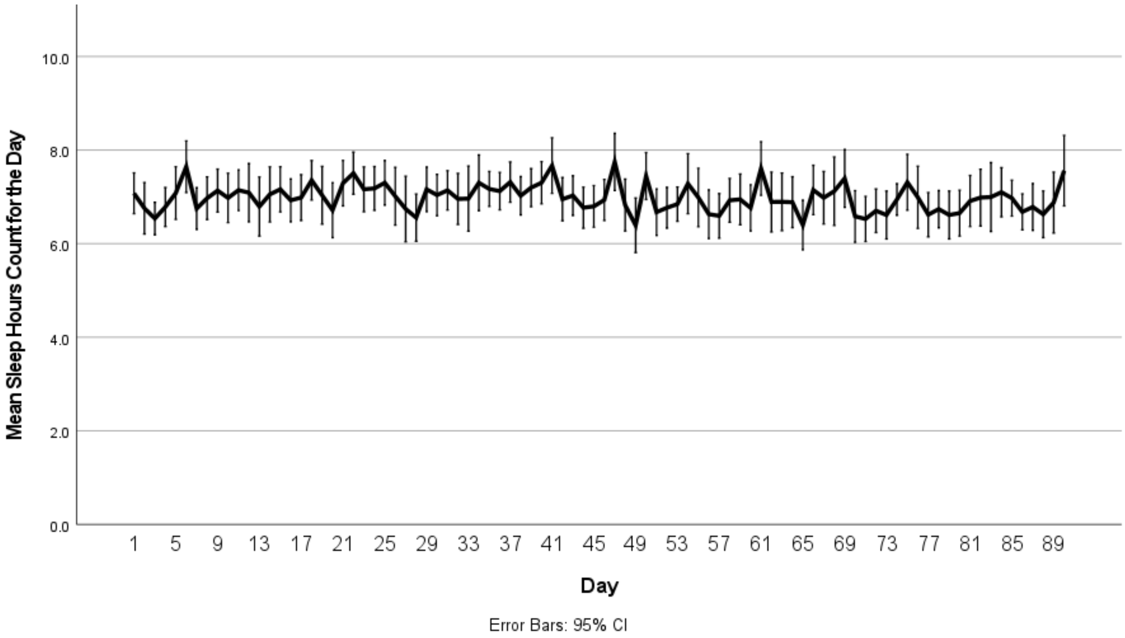

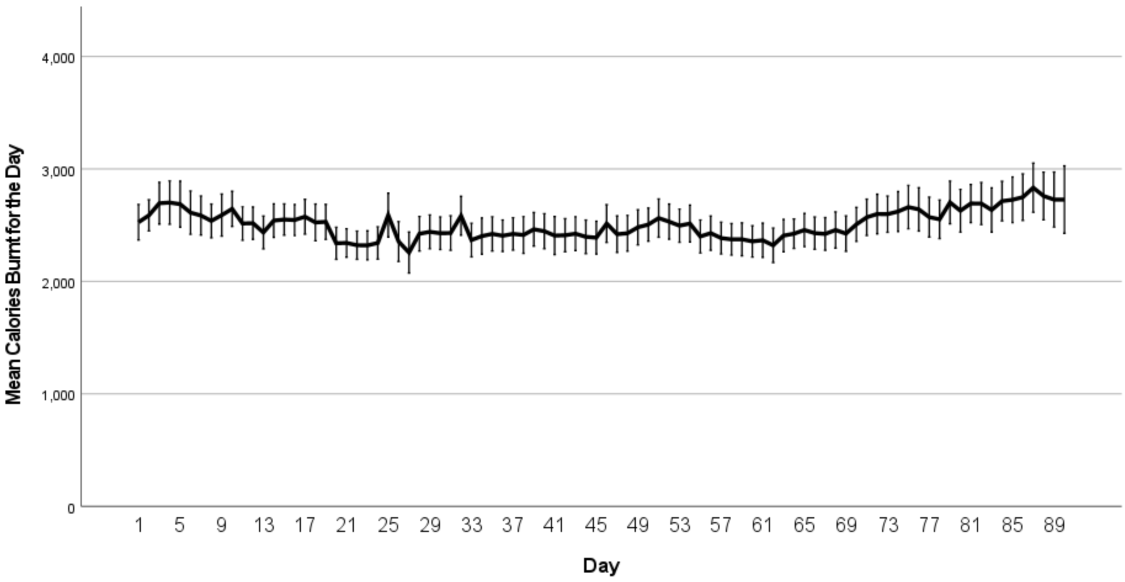

Error Bars: 95% CI

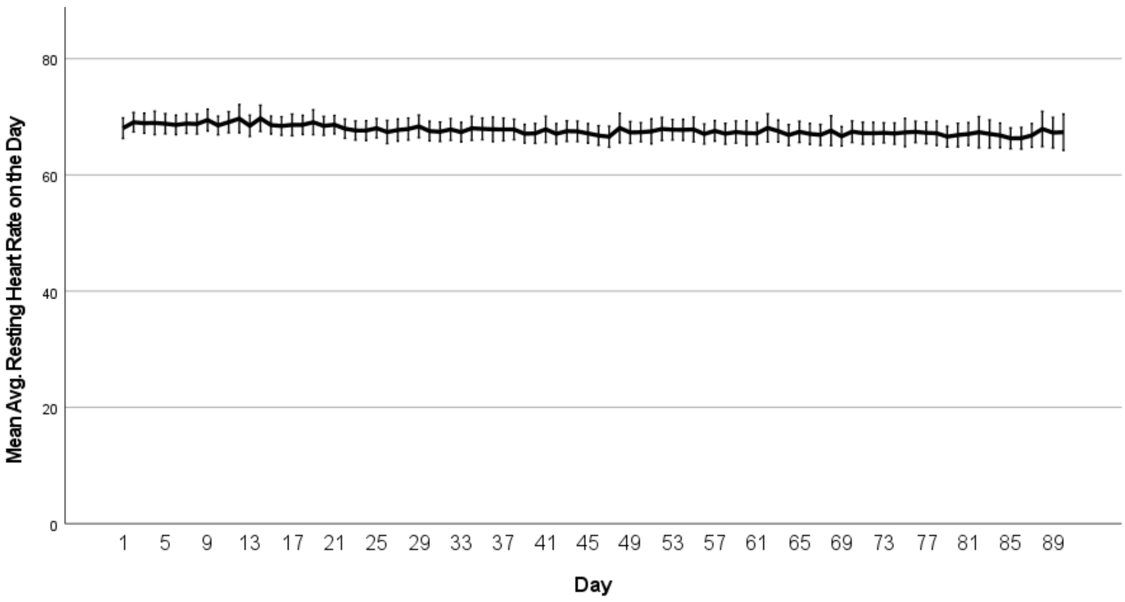

Error Bars: 95% CI
